# Hidradenocarcinoma: Five Years of Local and Systemic Control of a Rare Sweat Gland Neoplasm with Nodal Metastasis

**DOI:** 10.7759/cureus.2884

**Published:** 2018-06-26

**Authors:** Benazir Mir Khan, Muhammad Atif Mansha, Nasir Ali, Ahmed Nadeem N Abbasi, Syed Mustajab Ahmed, Bilal M Qureshi

**Affiliations:** 1 Oncology, Aga Khan University, Karachi, PAK

**Keywords:** hidradenocarcinoma, head and neck cancer, sweat gland neoplasm, radiation oncology, oncology, dermotology

## Abstract

Hidradenocarcinoma is a rare and locally aggressive tumor rendering a poor prognosis. Furthermore, very few cases present with nodal metastasis. Diagnosing such an entity, and then differentiating it from a benign counterpart, poses a great challenge to the clinicians. There are no established treatment guidelines for the management of this disease, particularly in patients with nodal involvement.

We present a case of a young male who was diagnosed with hidradenocarcinoma of the scalp, along with a neck swelling. A thorough diagnostic evaluation was done with endoscopy, pathological, and radiological investigations. He was successfully treated with resection of the scalp lesion and right-sided neck dissection followed by adjuvant concurrent chemoradiation. He remains free of any local and distant disease after five years of regular follow-up.

## Introduction

Hidradenocarcinoma is a rare and aggressive tumor that arises from the sweat glands. It is known for its tendency to recur locally and to metastasize to distant sites [1]. The management of this rare entity is not well-defined. Despite the use of multiple treatment modalities, the outcome still remains poor [2]. We describe the case of a man who had metastatic disease in the neck node at presentation.

## Case presentation

A 38-year-old male presented to the otorhinolaryngology clinic with the complaint of right-sided neck swelling in February 2013. This swelling had been progressively increasing for three months. There was no associated pain, fever, or difficulty in swallowing. He also reported having a painless swelling on the scalp which had been there for 20 years. On examination, there was a firm, fixed, non-tender mass palpable on the right side of the neck at level II. It measured 3 x 3 cm in size. Another lump was appreciated on the scalp, which was soft in consistency, non-tender, mobile, and 4 x 4 cm in size.

**Figure 1:**
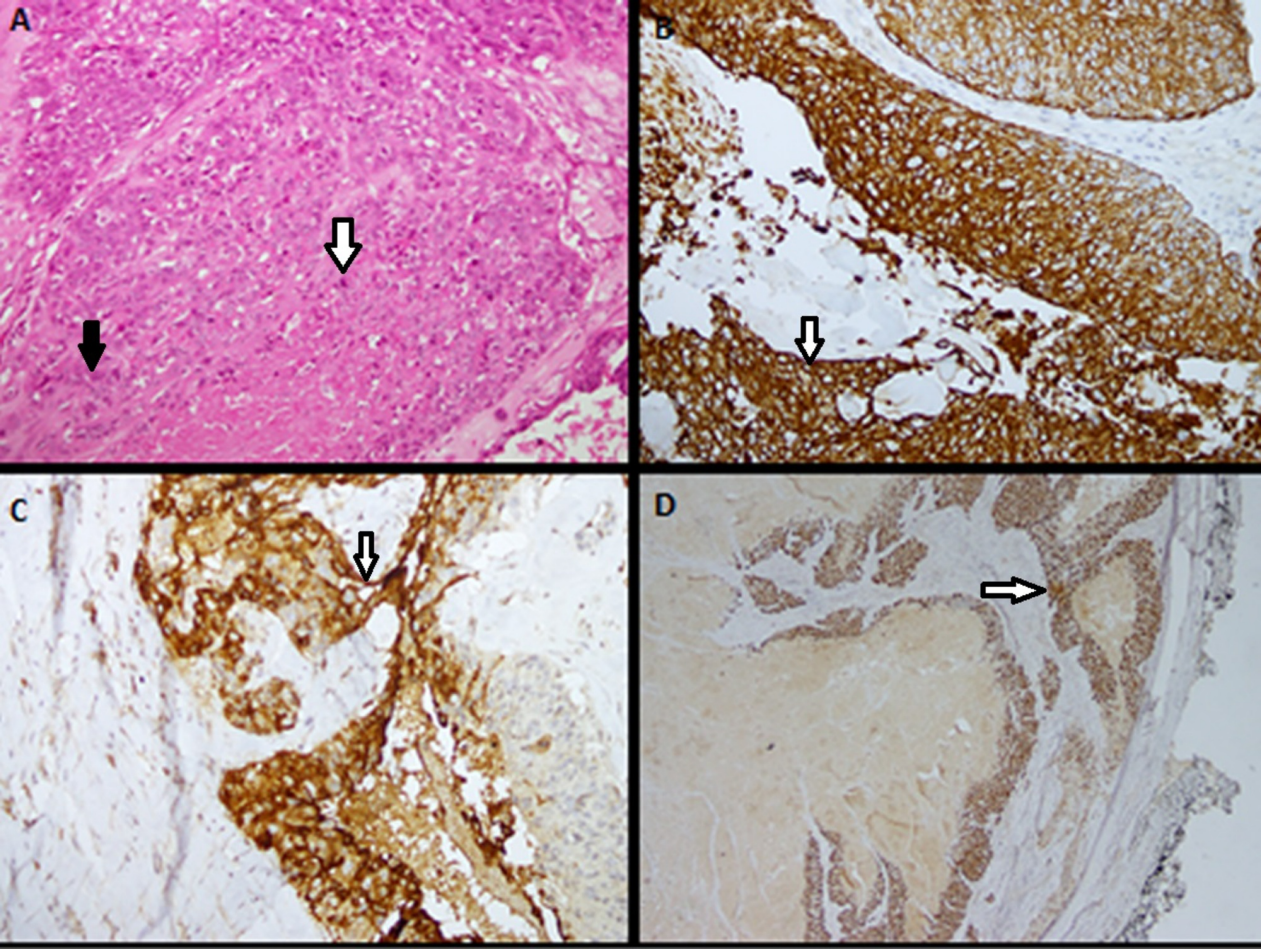
Immunohistochemical staining A: Sheets of tumor cells showing pleomorphic cells (black arrow) and frequent mitotic figures (white arrow) (H & E; 100x); B: Tumor cells showing positive staining with immunohistochemical stain cytokeratin 7 (white arrow); C: Tumor cells showing positive staining with immunohistochemical stain EMA (white arrow); D: Tumor cells showing positive staining with immunohistochemical stain p63 (white arrow). H&E: hematoxylin and eosin; EMA: epithelial membrane antigen

**Figure 2:**
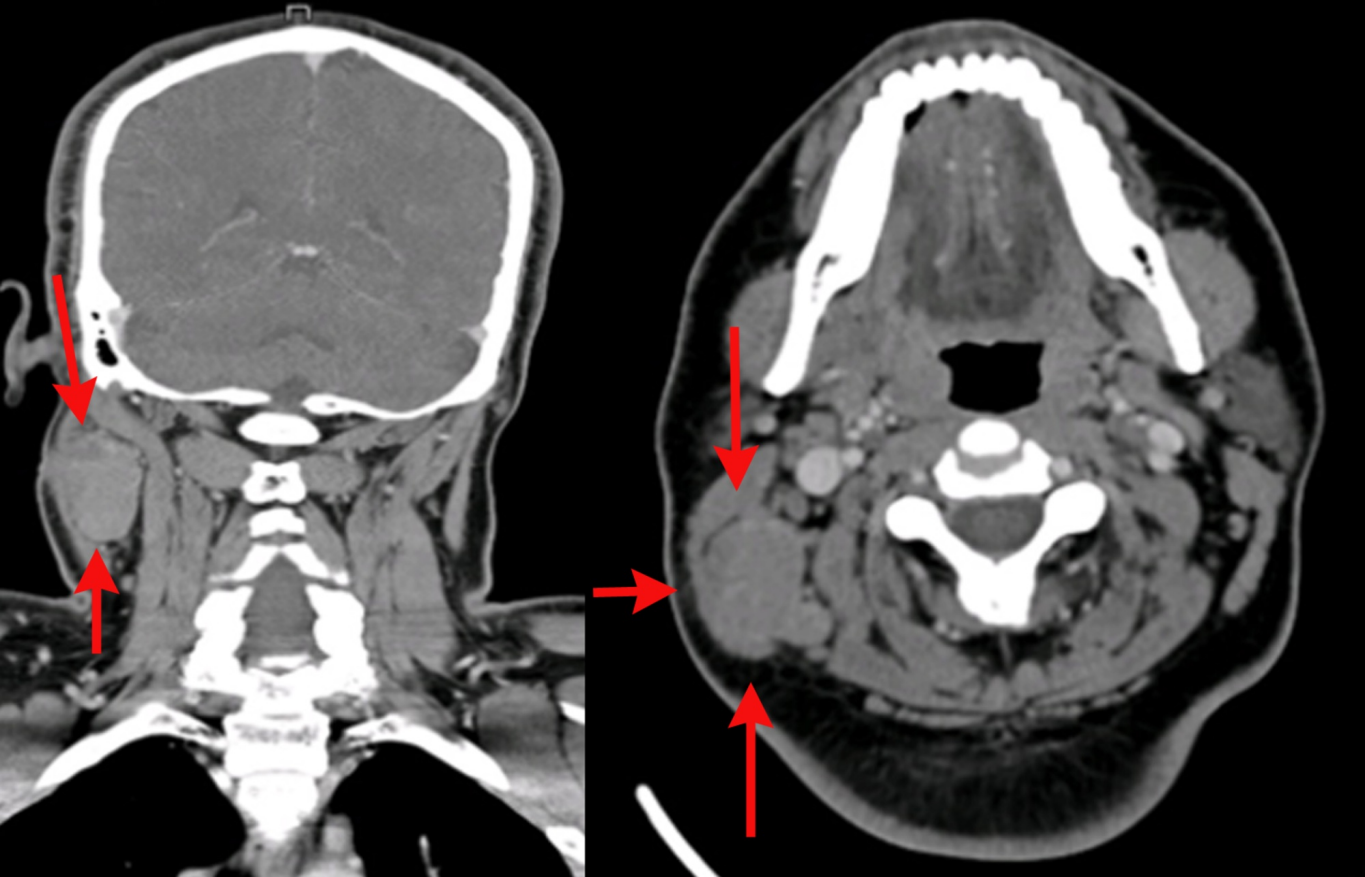
Computed tomography scan showing enlarged lymph node on right side of the neck (red arrows)

**Figure 3:**
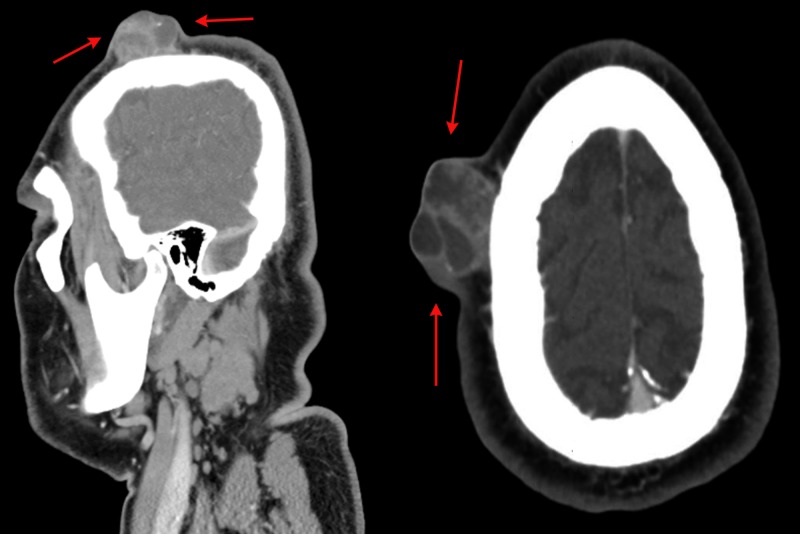
Computed tomography scan showing a lobulated mass lesion on the scalp (red arrows)

**Figure 4:**
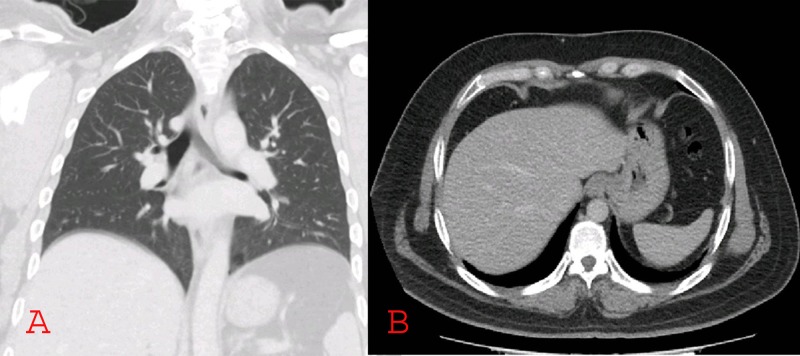
Computed tomography scan showing normal lungs (A) and liver (B)

Considering these clinical findings, he underwent excisional biopsy of the right nodal mass which suggested hidradenocarcinoma. Histopathologic evaluation revealed sheets of tumor cells showing pleomorphic cells and frequent mitotic figures. On immunohistochemical staining, tumor cells showed positivity for cytokeratin 7, epithelial membrane antigen (EMA), and p63 (Figure 1). A panendoscopy showed no abnormality in the pharynx, nasal cavity, or larynx. The locoregional extent of the disease was evaluated by a computed tomography (CT) scan of the head and neck that showed multiple enlarged lymph nodes on the right side of the neck (Figure 2), along with a well-defined lobulated cystic mass over the right side of the scalp (Figure 3). CT scans of the chest and abdomen were negative for any distant metastasis (Figure 4).

The case was discussed in the head and neck multidisciplinary tumor board meeting at our hospital. On the basis of the available evidence, the consensus was to go for a wide local excision of the scalp lesion, along with a right-sided neck dissection. The histopathology of the scalp lesion was reported as malignant hidradenocarcinoma. The size of the lesion was 4.2 x 3.5 x 2.2 cm with a closest resection (deep) margin of 0.1 cm. A total of 56 lymph nodes were recovered from the right side of the neck, out of which two were positive for tumor metastasis at level II, the largest deposit being 2.3 cm in size.

Postoperatively, the case was again discussed in the tumor board meeting where the consensus was to offer concurrent chemoradiation. Radiation therapy was offered in two phases to a total dose of 60 Gy in 30 fractions. In phase 1, a radiation dose of 50 Gy was given to the scalp and ipsilateral neck from level II to level IV. A boost dose of 10 Gy was delivered in phase 2 to the deep margin in the scalp and level II neck only. Concurrent cisplatin was given with the radiation at a dose of 100 mg/m^2^ as a radiosensitizer. After completion of treatment, he was followed up with clinical examination and serial imaging of the head and neck. Currently, he has completed five years of follow-up and is disease-free, both for local and distant metastasis.

## Discussion

Hidradenocarcinoma is a rare malignant neoplasm arising from the sweat glands of the skin [2]. It was first reported in 1948 in Brazil [3]. It accounts for 6% of malignant eccrine tumors, which in turn are found in 1:13,000 skin biopsies [4, 5]. Most common age of presentation is during the sixth and seventh decade with head, trunk, and extremities being the most common sites of involvement [6].

It is very difficult to diagnose and differentiate hidradenocarcinoma from its benign counterpart. Immunohistochemical analysis plays a very helpful role in diagnosing this entity. Hidradenocarcinoma shows strong positivity for Ki-67 and p53, for keratin AE1/AE3 and cytokeratin 5/6, and negativity for carcinoembryonic antigen (CEA), S-100 protein, gross cystic disease fluid protein 15 (GCDFP-15), EMA, and bcl-2 [7].

A five-year disease-free survival rate of less than 30% has been reported in the literature, suggesting a poor prognosis [8]. The best treatment modality is not clear yet as the literature is limited. Contemporary data suggest that surgical excision has been widely used as the definitive treatment. In 2004, Japanese surgeons reported the case of a young woman who had a scalp mass of 10 months duration. Her metastatic workup was negative. She underwent resection of the mass under local anesthesia without any further treatment. No recurrence was reported until two years of follow-up [5].

The natural history of this aggressive tumor mandates the use of adjuvant treatment after surgery for better local control. Lalya et al. highlighted the role of adjuvant radiation therapy in a case with recurrent hidradenocarcinoma in the parotid gland region. Adjuvant radiation with a dose of 66 Gy and 50 Gy was delivered to the tumor bed and regional lymphatic chains, respectively. After a follow-up of more than 15 months, the patient had local control of the disease without significant toxicity [9]. The role of adjuvant treatment in a metastatic setting was explored by Souvatzidis et al., who presented a series of seven cases diagnosed with malignant nodular hidradenoma. Out of the seven patients, six had lymph node metastasis and were offered adjuvant treatment in the form of either chemotherapy or radiation therapy. Only one patient received concurrent chemoradiation therapy and had a survival of 45 months, which was much better than the others [2].

The validity of chemotherapy use in this rare tumor is imperceptible because of very few case reports. An article from Tunisia highlighted the case of a 52-year-old woman who was diagnosed with hidradenocarcinoma on the ring finger of left hand, for which surgical excision was performed. Four years later, the lesion recurred with multiple cutaneous metastases along the left arm. She was given four cycles of chemotherapy with 5-fluorouracil but the disease remained stable. Second-line chemotherapy was given with a combination of doxorubicin and cisplatin. After four cycles, the evaluation revealed a partial response [10]. Jouary et al. treated a patient with recurrent metastatic hidradenocarcinoma. The tumor recurred many times after surgical and electron-beam treatments. First-line chemotherapy with carboplatin failed. Capecitabine was then started as a second line treatment. After 12 cycles of chemotherapy, more than a 50% tumor reduction was noted [11].

In our patient, a wide local excision with ipsilateral neck dissection, followed by concurrent chemoradiation was used; we found good local and systemic control up to this point. That being said, in order to develop a standard of care, more literature support is required.

## Conclusions

Hidradenocarcinoma is a rare malignancy that should be addressed with an aggressive trimodality approach. A multidisciplinary approach in our patient has produced a curative outcome. High-quality clinical research evidence to guide treatment decisions is lacking, and there is need to identify and evaluate new treatment strategies.
